# A Novel Aqueous Two Phase System Composed of a Thermo-Separating Polymer and an Organic Solvent for Purification of Thermo-Acidic Amylase Enzyme from Red Pitaya (*Hylocereus polyrhizus*) Peel

**DOI:** 10.3390/molecules19056635

**Published:** 2014-05-22

**Authors:** Mehrnoush Amid, Yazid Manap, Nor Khanani Zohdi

**Affiliations:** Department of Food Technology, Faculty of Food Science and Technology, Universiti Putra Malaysia, 43400 UPM Serdang, Selangor, Malaysia

**Keywords:** novel aqueous two phase system, thermo-separating copolymer, organic solvent, thermo-acidic amylase, recycling of phase components, partition coefficient, purification fold, yield

## Abstract

The purification of thermo-acidic amylase enzyme from red pitaya (*Hylocereus polyrhizus*) peel for the first time was investigated using a novel aqueous two-phase system (ATPS) consisting of a thermo-separating copolymer and an organic solvent. The effectiveness of different parameters such as molecular weight of the thermo-separating ethylene oxide-propylene oxide (EOPO) copolymer and type and concentration of organic solvent on the partitioning behavior of amylase was investigated. In addition, the effects of phase components, volume ratio (V_R_), pH and crude load of purification factor and yield of amylase were evaluated to achieve the optimum partition conditions of the enzyme. In the novel ATPS method, the enzyme was satisfactorily partitioned into the polymer-rich top phase in the system composed of 30% (*w/w*) EOPO 2500 and 15% (*w/w*) 2-propanol, at a volume ratio of 1.94 and with a crude load scale of 25% (*w/w*) at pH 5.0. Recovery and recycling of components was also measured in each successive step of the ATPS process. The enzyme was successfully recovered by the method with a high purification factor of 14.3 and yield of 96.6% and copolymer was also recovered and recycled at a rate above 97%, making the method was more economical than the traditional ATPS method.

## 1. Introduction

An aqueous two-phase system (ATPS) method has been proposed as an ideal purification technique for the separation and recovery of biomolecules because of the system’s high productivity, simplicity, short processing time, cost effectiveness, scalability and versatility [1,2]. Therefore, ATPS has been widely employed for the separation of biological materials such as cells, cell debris, proteins and nucleic acids [3]. This method could be used to overcome the problem faced by conventional purification methods because of its speed, low interfacial tension, easy scale-up, minimized protein denaturation, non-toxicity and mild conditions [4]. It should be noted that the most well-known form of the method is based on two polymers e.g., polyethylene glycol (PEG) and dextran or one polymer and salt (e.g., PEG/sodium citrate) which have some disadvantages such as low segregation, high cost of polymers (e.g., dextran) and the complication of polymer isolation from the purified biomolecules [5]. A novel ATPS method using a thermo-separating polymer and organic solvent overcomes this drawback of the traditional ATPS methods. This system makes it possible to create two phases and the polymer rich top phase and organic solvent bottom phase can be recycled [6]. Thus, the improved ATPS method can minimize the overall cost and the separation process of target proteins from phase solution will be simplified. Furthermore, recycling the solution components can minimize environmental pollution. Amylases represent one of the most important industrial enzymes and have a wide variety of applications in different industrial fields, such as the food, fermentation, textile, paper, detergent, pharmaceutical and sugar industries [7]. These enzymes account for about 30% of the World’s enzyme production [8]. Recent advances in plant biotechnology have enabled plants to be used as a bioresource to produce novel enzymes for industrial and biotechnological applications [9]. During processing of pitaya, some byproducts such as peels are generated. Pitaya peel is mostly a waste material from fruit and beverage industries. Although it constitutes about 33% of whole fruit weight and possesses valuable kinds of enzymes such as amylase, it is not currently being utilized commercially in any way, and ends up as a waste product [10], but it could be beneficially utilised for the commercial and economical production of natural enzymes. There is no information regarding the recovery of amylase from red pitaya peel using the application of such a recyclable ATPS. In this study, the feasibility of recovering thermo-acidic amylase and recycling the phase components in a novel ATPS composed of a thermo-separating polymer and organic solvent was investigated for the first time. The partitioning efficiency of amylase in the ATPS and the effects of the molecular weight of thermo-separating copolymer, type and concentration of organic solvent, volume ratio, crude load as well as pH were studied to achieve a high purification factor and yield of the amylase enzyme. In this study, the recycle recovery of the thermo-separating copolymer at each recycling step was also investigated. 

## 2. Result and Discussion

### 2.1. The Influence of Phase Components on the Amylase Activity

The extracted enzyme was mixed with thermo-separating polymer and organic solvents to investigate the effect of the phase components on the amylase activity. The results in Table 1 show that there are significant (*p* < 0.05) differences in the amylase activity for different EOPO molecular weights. In fact, the thermo-separating copolymer showed different degrees of hydrophobicity with different EOPO content. EOPO with low PO content (*i.e.*, 2,500) was more suitable for the amylase partitioning compared to other EOPO molecular weights. It could be considered that EOPO 2500 with low PO content has better effect on the enzyme partitioning because it enables maximal solubility of the amylase polymer phase and subsequently the amylase precipitation in the interphase could be avoided [11]. Thus, the highest amylase activity was achieved in the presence of 30% (*w/w*) of EOPO 2500, while EOPO with higher molecular weight significantly (*p* < 0.05) decreased the amylase activity. One reason for this could be that the surge in polymer MW increases the exclusion effect. As such, the polymer adopts a more compact conformation with intermolecular hydrophobic bonds and thus prevents the presence of biomaterial in the top phase. As a result, there would be significant target protein transfer to the organic solvent-rich bottom phase. According to our preliminary studies, the enzyme showed the high activity and stability in the presence of organic solvent (*i.e.*, 1-propanol, 2-propanol and ethanol). It is believed that organic solvent helps to maintain the open confirmation of the enzyme through exposure of the active site crevice, to simulate amylase activity [12]. In the study, the amylase showed the highest activity in solution of 1-propanol and 2-propanol rather than ethanol (Table 1). This phenomenon could be due to the longer hydrophobic chain of propanol compared to ethanol and so the interaction of the amylase and propanol was more facile and this led to an increase in the enzyme partitioning. Therefore, the highest amylase activity was obtained in the presence of 10% (*w/w*) of 2-propanol, while the enzyme activity was significantly (*p* < 0.05) decreased in the presence of ethanol. The results showed that the higher concentration of organic solvent has a negative effect on the enzyme activity, which could be due to denaturation of the enzyme in the condition. After reviewing the results presented in Table 1, the EOPO 2500/2-propanol system was chosen for further optimization. 

**Table 1:** Effect of EOPO molecular weight and organic solvent on amylase activity.

Phase composition		Concentration (%)		Relative activity	
EOPO2500		10		101.1 ± 1.33 ^a^	
		30		130.4 ± 0.21	
		50		103.2 ± 0.01 ^e^	
		70		100.2 ± 0.00 ^a^	
EOPO 3900		10		99.3 ± 0.11 ^a^	
		30		97.1 ± 0.21 ^a^	
		50		101.2 ± 0.32 ^d^	
		70		98.6 ± 0.57 ^ab^	
EOPO 12000		10		83.2 ± 0.09 ^f^
		30		78.2 ± 1.42 ^g^
		50		66.3 ± 0.20 ^g^
		70		67.1 ± 0.32 ^h^
Ethanol		10		97.2 ± 0.12 ^a^
		30		88.1 ± 0.07 ^e^
		50		86.6 ± 0.10 ^e^
		70		83.2 ± 0.52 ^b^
1-propanol		10		100.2 ± 0.22 ^a^
		30		126.1 ± 0.07 ^e^
		50		118.6 ± 1.10 ^e^
		70		98.2 ± 0.52 ^b^
2-propanol		10		140.2 ± 1.23 ^i^
		30		115.2 ± 0.16 ^d^
		50		121.6 ± 0.02 ^e^
		70		101.6 ± 0.0 ^a^

The results reported are the mean of triplicate readings with an estimated error of ±10% (mean ± SD). ^a–g^ Mean value followed by different letters differs significantly (*p* < 0.05).

### 2.2. Optimization of EOPO/2-Propanol ATPS

To optimize the amylase partition efficiency in EOPO2500/2-propanol system, 16 systems were evaluated. These systems systematically varied the 2-propanol and EOPO concentrations. According to Table 2, partitioning of the amylase was significantly (*p* < 0.05) enhanced in 30% (*w/w*) EOPO 2500 and 15% (*w/w*) 2-propanol, which showed a purification factor of 12.6 and a yield of 94.8% (Table 2). Results indicate that amylase partitioning is better at lower concentrations of 2-propanol and EOPO 2500. In general, increasing the EOPO concentration decreases the partition efficiency [13]. A similar trend was observed when the copolymer concentration was increased. This was due to the gradual dehydration of the bottom phase as the EOPO concentration in the top phase increased, thus causing an imbalance which did not favour amylase retention in the top phase. High concentrations of organic solvent in the bottom phase might have a negative effect on the activity of the enzyme and cause denaturation of the amylase, thus, decreasing the partitioning efficiency of the system [14]. Therefore, the composition of 30% (*w/w*) EOPO 2500 and 15% (*w/w*) 2-propanol was selected for the future studies.

#### 2.3. Phase Diagram of EOPO Copolymer/Organic Solvent ATPS

**Figure 1:**
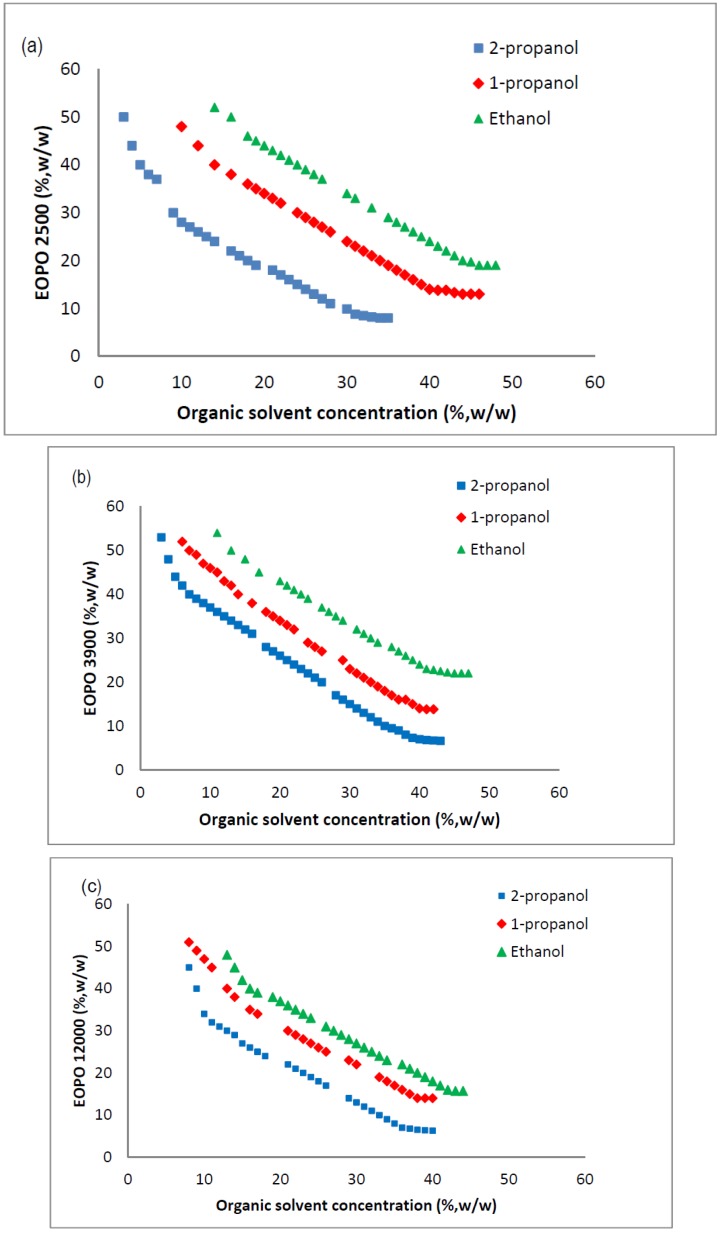
Phase diagrams (binodal curves) of EOPO copolymers/organic solvents ATPSs; The binodal curves for ethanol (▴), 2-propanpol (■) and 1-propanol (♦) were plotted against (**a**) EOPO 2500, (**b**) EOPO3900 and (**c**) EOPO 12000.

The phase diagrams (binodal curves) of various copolymers (EOPO 2500, EOPO3900 and EOPO 12000) with the three types of organic solvents (ethanol, 1-propanol and 2-propanol), are shown in Figure 1a–c. The binodal curves associated with the EOPO/ethanol systems were further from the origin than the EOPO /propanol-system curves, indicating that ethanol’s higher polarity improved the overall solubility and miscibility of the salts. 

**Table 2:** Partition of amylase in different concentration EOPO 2500/2-propanol systems

Mixing Point		Purification Factor	Yield (%)
EOPO 2500 (%,*w/w*)	2-Propanol (%,*w/w*)		
10	15	8.5 ± 0.12 ^a^	50.4 ± 0.15 ^a^
10	35	8.8 ± 1.32 ^a^	51.2 ± 0.08 ^a^
10	55	9.3 ± 0.81 ^b^	62.3 ± 0.04 ^b^
10	75	9.7 ± 0.52 ^b^	74.1 ± 0.12 ^c^
30	15	12.6 ± 0.01 ^c^	94.8 ± 0.13 ^d^
30	35	10.2 ± 0.11 ^d^	93.6 ± 0.01 ^e^
30	55	10.3 ± 0.05 ^c^	72.5 ± 0.02 ^c^
30	75	9.2 ± 0.14 ^b^	64.2 ± 0.10 ^b^
50	15	8.4 ± 0.08 ^a^	73.2 ± 0.02 ^c^
50	35	7.9 ± 0.13 ^e^	62.1 ± 0.11 ^b^
50	55	7.2 ± 0.14 ^e^	60.5 ± 0.12 ^b^
50	75	6.8 ± 0.21 ^e^	52.8 ± 0.22 ^a^
70	15	5.8 ± 0.07 ^f^	43.1 ± 0.01 ^f^
70	35	5.2 ± 0.01 ^f^	40.2 ± 0.22 ^f^
70	55	3.6 ± 0.17 ^h^	37.3 ± 0.09 ^g^
70	75	1.2 ± 0.04 ^i^	33.5 ± 0.06 ^g^

The results reported are the mean of triplicate readings with an estimated error of ±10% (mean ±SD). ^a-g^ Mean value followed by different letters differs significantly (*p* < 0.05).

Amongst the EOPO/organic solvent systems, the binodal curves of the EOPO/2-propanol systems were closer to the origin than the binodal curves of EOPO/1-propanol systems. This phenomenon was attributed to 1-propanol’s higher hydrophobicity. Higher hydrophobicity causes poor salt solubility and miscibility; thus, a lower concentration of phase components is needed to form the two-phase system. 

It should be noted that the binodal curve observed in the EOPO12000/ethanol phase diagram was shorter than the other curves, indicating that the ATPS was probably unstable under this condition. This may be due to the low solubility of EOPO 12000 in the ATPS, thus, component precipitation could easily have occurred.

#### 2.4. Influence of Crude Load on Amylase Partition Behavior

The application of the novel ATSP was performed using various crude loads from 15% up to 55% (*w/w*). Table 3 shows the influence of crude load on the amylase partitioning. It should be noted that the concentration of crude loading plays an important role in the enzyme partitioning. The crude feedstock components could change the partitioning behavior of the protein. In addition, the loaded crude feedstock could alter the phase ratio of the system and influence the purification factor of the enzyme [15,16]. An increment in the amounts of the amylase and contaminants in the system could decrease ATPS performance. Therefore, increasing the crude load up to 25% caused a decrease in the partitioning of the amylase (Table 3). However, the partitioning was increased with an increasing crude load concentration from 15% to 25% (*w/w*) and a crude feedstock load of 25% (*w/w*) showed the maximum capacity, based on 10 g of ATPS. Under these conditions, purification and selectivity were 8.06-fold and 85.6-fold, respectively. Therefore, it is clear that 25% (*w/w*) sample loading should be feasible to obtain maximum top phase recovery of the amylase from the crude extract

**Table 3:** Effect of crude load on selectivity and yield of amylase partitioning.

Crude load (%,*w/w*)	Tie line length	Purification fold	Yield (%)
15	36.4	2.31 ± 1.2 ^a^	74.3 ± 0.5 ^a^
25	40.1	8.06 ± 0.5 ^b^	85.6 ± 0.2 ^b^
35	48.3	4.07 ± 1.1 ^c^	63.4 ± 1.1 ^c^
45	52.3	3.02 ± 1.3 ^c^	55.3 ± 0.3 ^d^
55	69.2	2.8 ± 2.2 ^c^	48.2 ± 0.4 ^d^

The results reported are the mean of triplicate readings with an estimated error of ±10% (mean ± SD). ^a-d^ Mean value followed by different letters differs significantly (*p* < 0.05).

#### 2.5. Influence of Volume Ratio on Partitioning of Amylase

**Figure 2:**
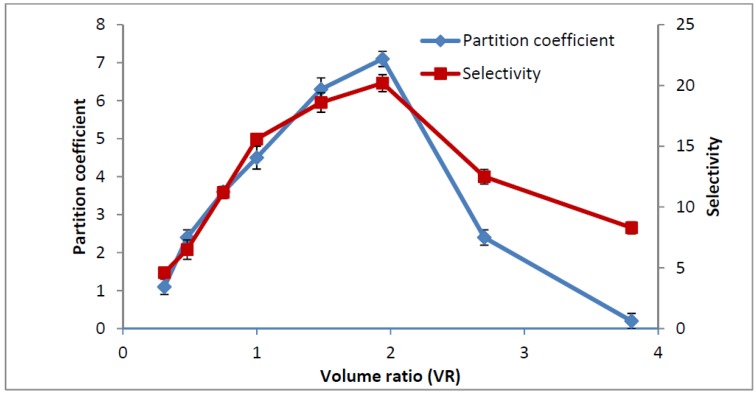
Effect of volume ratio on partition coefficient and selectivity of amylase.

Figure 1 shows the effect of volume ratio on the amylase partitioning. The partitioning of the amylase showed that the highest partitioning coefficient and purification factor of the amylase was obtained at 1.94 volume ratio. It is believed that as the volume ratio increases, the protein partition coefficient increases as more target protein is partitioned into the polymer-rich top phase. This could be explained by the fact that that the protein partitioning into the top phase increased with an increase in volume ratio mainly due to an increment in the volume of the top phase. It means that with a higher volume ratio the available free space to accommodate the enzyme in the top phase is bigger. Therefore, a higher degree of partitioning of the enzyme in the top phase results in a higher volume ratio of the system. This phenomenon could be due to an increase of the hydrophobic interaction between the target enzyme and co-polymer that led to the desirable partitioning or transfer of the target enzyme (amylase) into the top co-polymer-rich phase at the certain volume ratio. The organic solvent bottom phase also affects the hydrophobic interactions between the co-polymer and proteins. The organic solvent ions at this volume ratio could be interacting with the oppositely charged groups of the protein, resulting in dehydration of the proteins. Thus, the hydrophobic interaction between dehydration target proteins and the co-polymer phase would be improved and lead to transfer of the amylase enzyme to the top phase. In the volume ratio, the top phase was occupied by target enzyme (amylase enzyme), thus, hydrogen binding interactions between other proteins (contaminants/unwanted proteins) and organic solvent could increase the transfer of contaminants into the bottom phase. Therefore, the activity of amylase in the top phase and protein concentration of bottom phase were increased at the volume ratio of 1.94. In the other hand, the activity of amylase in the bottom phase and protein concentration of the top phase were decreased under these conditions. It could be confirmed that a certain and optimized volume ratio point in the system provided suitable conditions to transfer the target enzyme to the top phase by hydrophobic interaction with the co-polymer-rich phase and also moved the contaminants by hydrogen bond interactions with the organic solvent of the bottom phase. In contrast, a lower phase volume ratio may cause precipitation of the enzyme at the interphase when the limit of the enzyme solubility in the EOPO phase is reached. Thus, loss of the amylase by precipitation could be minimized at higher volume ratios. It should be noted that a markedly increased volume ratio in the system could also have a negative effect on the enzyme partitioning due to greater dilution of the amylase concentrations in the system, thus decreasing the efficient partitioning of the enzyme into the desired phase [17]. Therefore, the optimization to find the best volume ratio for the proper partitioning of the enzyme should be investigated in ATPS. According to the results, the partition coefficient and selectivity of V_R_ = 1.94 were 7.3 and 19.2, respectively (Figure 2). Thus, the results indicated that the ATPS with V_R_ of 1.94 gave a higher purification of amylase and was therefore optimum for amylase partitioning.

#### 2.6. Influence of pH on Amylase Partitioning

**Figure 3:**
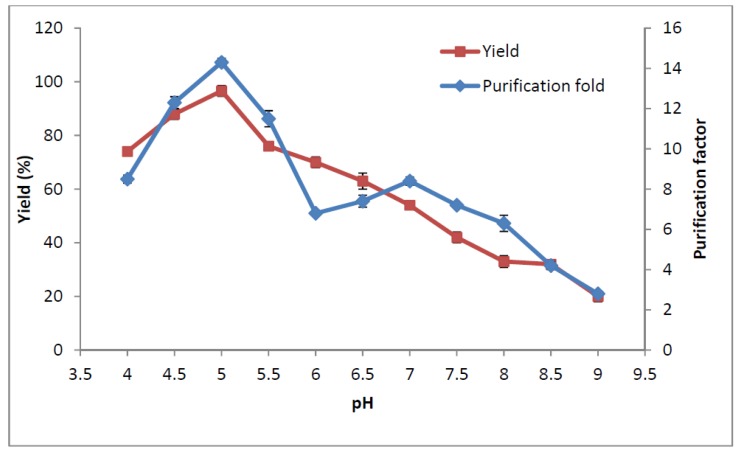
Influence of pH on purification factor and yield of amylase.

The effect of pH on the purification factor and yield of the amylase is shown in Figure 3. Partitioning is affected by pH when the charge of the solution or the ratio of the molecules charge is changed. It appears that pH modifies the partitioning of the target protein and changes the concentration of the phase-forming polymer due to the lessening or addition of charges on the functional groups of polymer. These changes could affect the repulsion or attraction between target proteins and polymer [18]. There are some reports which show that negatively charged biomolecules prefer to partition into the top phase at higher pH, thus, the partition coefficient was increased by electrostatic interactions between the protein and polymer phases [19]. It should be noted that the ion composition of amylase and the surface properties of the protein contaminants will be changed in various pH in ATPS, which could affect the partitioning properties of the enzyme [20]. The amylase from pitaya peel at pH 5.0 that is near to its isoelectric point, pI 4.7, was shown to be a negatively charged protein, with a tendency to partition into the top polymer-rich phase and interact with EOPO polymer molecules. This change in the partitioning behaviour of the enzyme was due to the changes in the protein charge. At pH values lower than 5.0, amylase was observed to be slightly positively charged and it partitioned into the bottom organic solvent phase. It should be pointed out that a negative effect was observed on the enzyme partitioning by increasing the pH above 5.0 (*i.e.*, pH 6.0 to 9.0). This would probably increase the electrostatic interaction of protein contaminants at a higher pH so that more contaminants move to the top phase and consequently, electrostatic interaction between amylase and polymer in the top phase is limited. Therefore, the highest purification factor 14.3 and yield 96.6% of the amylase partitioning were achieved at pH 5.0 and this pH 5.0 was selected as the optimum for the recovery of the amylase. 

#### 2.7. Recycling of Temperature Induced Top Phase Polymer

**Figure 4:**
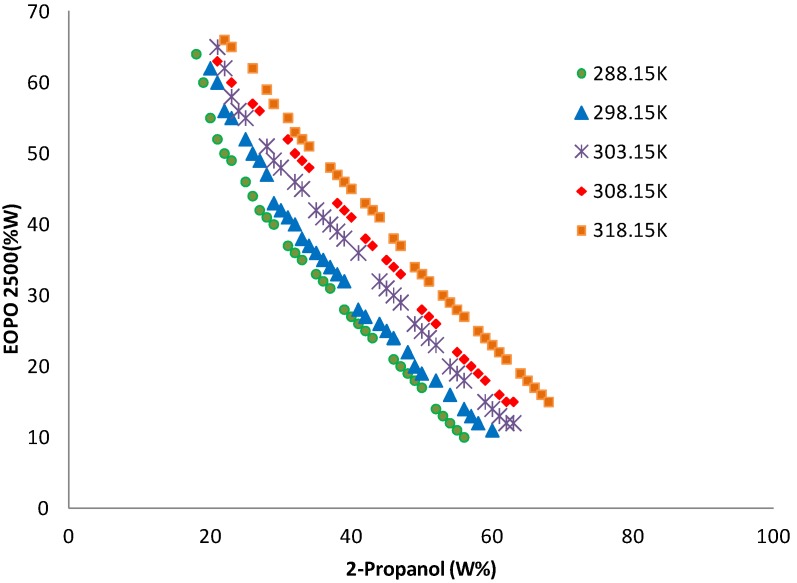
The phase equilibria of the optimum recycling system containing EOPO 2500 and 2-propanol at different temperature. (●) 288.15K,(▴) 298.15K,(×) 303.15K, (♦) 308.15K and (■) 318.15K.

**Figure 5:**
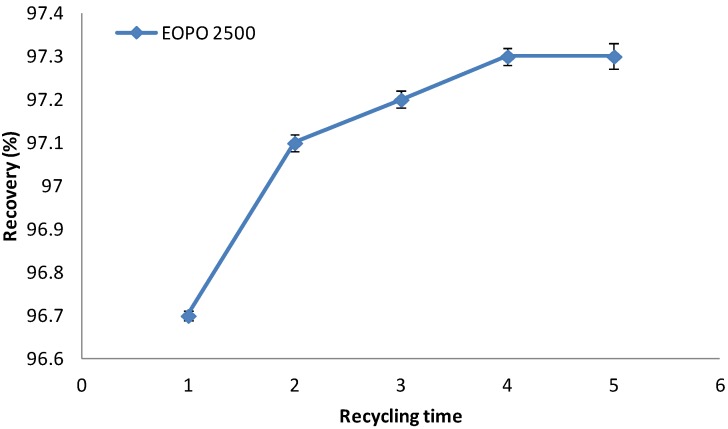
The recycle recovery of EOPO 2500 copolymer.

Figure 4 indicates that behavior of EOPO copolymers at various temperatures. Figure 5 shows the total recovery of EOPO copolymer at each recycling step. However, the EOPO 2500 recoveries were almost similar as indicated in Figure 5. The results showed only minor loss of the polymer in the recycling steps whereas the recovery of copolymer could still be maintained over 97% after five cycles in relation to the initial amount. This could be explained by the good ability of the copolymer to take desirable protein into the top phase after being reused several times. The copolymer reached its maximum capacity to accommodate the negatively-charged protein, therefore the protein newly-dispensed into the ATPS could be partitioned into the copolymer-rich top phase [21]. This new system can reduce the cost significantly, and shows great potential for application in industry.

#### 2.8. Amylase Recovery

**Figure 6:**
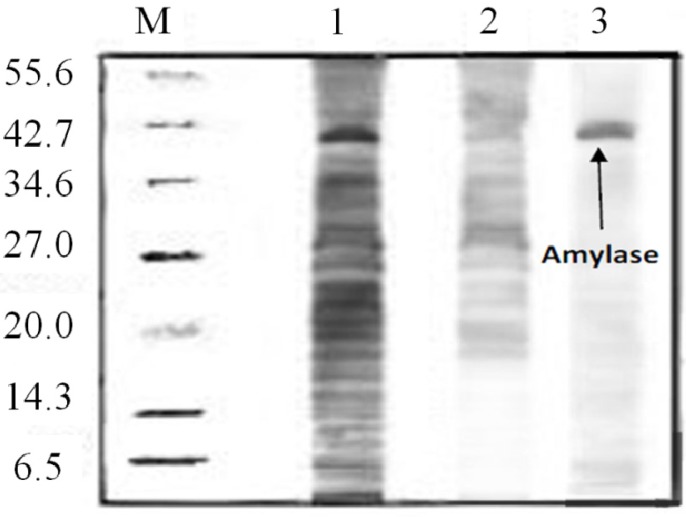
The purity of the partitioned amylase was assessed by SDS-PAGE analysis. Lanes: M= protein molecular markers; 1 = crude feedstock; 2 =ATPS bottom phase lane; 3 = ATPS top phase.

The purity of the amylase from the first recovery and the top phase from the recycling steps was investigated employing 12% sodium dodecyl sulfate gel electrophoresis (SDS-PAGE, Figure 6). As shown in Figure 6. Lane 1 was identified as crude feedstock with lots of impurity bands. Line 2 contained the sample of the aqueous phase, which indicates lesser, and fainter bands than crude feedstock. The sample recovered from the top phase indicated just one dark band at 42.3 kDa (Lane 3). It should be noted that the amylase activity (Section 3.6) and protein concentration of the sample (Section 3.7) were determined before loading of sample into SDS-PAGE to verify that the enzyme was amylase. The results showed high activity of amylase (628U) and low protein concentration (0.12 mg/mL), which confirmed that the enzyme was amylase.As such, this SDS-PAGE result indicated the efficacy of the purification technique in this study which gives maximum recovery of amylase from red pitaya peel.

## 3. Experimental

### 3.1. Chemicals and Plant Material

All chemicals and reagent were analytical grade. Bradford Reagent, bovine serum albumin (BSA), 3,5-dinitrosalicylic acid (DNS) were obtained from Sigma Chemical Co., (St. Louis, MO, USA). Sodium acetate, acetic acid, sodium citrate, citric acid, soluble starch, sodium potassium tartrate (NaKC_4_H_4_O_6_∙4H_2_O) was obtained from Merck (Darmstadt, Germany). Red pitaya fruits (*Hylocereus polyrhizus*) were purchased from Passer Brong (Selangor, Malaysia). Ripened pitaya fruits were selected based on the size uniformity at the same stage of ripening and free of visual defects. The fruits were stored in a cold room at 4 °C until using for extraction procedure.

### 3.2. Extraction of Amylase

Fresh pitaya fruits (2 Kg) were cleaned and rinsed thoroughly with sterile distilled water and dried with tissue paper. The pitaya peels were removed and chopped into small pieces (1 cm^2^ each, 1 mm thick), and then they were quickly blended for 2 min (Model 32BL80, Dynamic Corporation of America, New Hartford, CT, USA) with sodium acetate buffer at pH 5.0 with a buffer to sample ratio of 4:1 at a temperature of 2.5 °C. The peel-buffer homogenate was filtered through cheesecloth, and then, the filtrate was centrifuged at 6,000 rpm for 5 min at 4 °C. The supernatant was collected [22]. The supernatant (crude enzyme) was kept at 4 °C for one overnight to be used for the purification step.

### 3.3. Preparation of Thermo-Separating ATPS

The ATPSs were prepared from EOPO stock solutions of different molecular weights and different concentration of organic solvent (50%, *w/w*). Preparation of phase systems was done in 15 mL graduated centrifuge tubes by weighing an appropriate amount of stock solution of EOPO and organic solvent with 20% (*w/w*) of crude enzyme extract. The different concentrations of copolymers and organic solvent were selected and a sufficient amount of distilled water was added to the system to obtain a final mass of 10 g system. Subsequently, the solutions were mixed gently by a vortex mixer before centrifugation at 4,000 ×*g* for 10 min in order to complete the phase separation. Finally, the volumes of both phases were measured and samples were removed and collected for future analysis by enzyme activity and protein determination assay. To minimize interferences of polymer and organic solvent, the controls of each system were prepared by adding 20% (*w/w*) distilled water instead of enzyme.

### 3.4. Preparation of Phase Diagram (Binodal Curve)

Several ATPS were prepared by mixing EOPO copolymer and organic solvent solution in each tube due to preparation of binodal curves. The mixture was initially turbid, indicating that two phases would eventually form. Distilled water was then added drop by drop, and each drop was followed by gentle mixing, until the turbidity disappeared. The phase-transition points were approximated by measuring the total weight of the added distilled water. The concentrations of the phase-forming components found in the final system were calculated. The binodal curve was then plotted at varying EOPO copolymer and organic solvent concentrations.

### 3.5. Recycling of Copolymer

Organic solvent-bottom phase from the amylase recovery process was collected and reused in subsequent ATPS runs due to the recycling phase component procedure. Meanwhile, distilled water was added in a ratio of 1:1 to dilute the EOPO copolymer top phase of the amylase recovery ATPS. This was followed by incubating the diluted top phase sample in a water bath at 65 °C (as lower critical solution temperature for EOPO 3900) for 15 min to achieve the thermo-separation [8]. Centrifugation of the diluted top phase sample at 4,000 rpm for 10 min followed to achieve total separation, after which the water top phase and EOPO bottom phase were separately withdrawn to complete the first recycling of the phase components. The next step was to weigh the EOPO bottom phase and to record the weight and determine polymer recovery. The concentrations of the polymer recovered were measured in refractive index by using refractometer and the calculation of the recovered polymer was carried out as shown in Equation (1) below [23]:

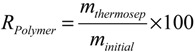
(1)
where, m_ thermosep _indicates the mass of recovered polymer in the bottom enriched polymer phase after the thermo-separation procedure and m_initial _is the total mass of polymer in the system. 

### 3.6. Amylase Activity Assay

Amylase activity in purified samples from pitaya peel was assayed by the dinitrosalycylic acid method of Kammoun [24] with some modifications. Purified enzyme was incubated with 150 µL soluble starch (25% *w/v*) prepared in sodium acetate buffer (pH 5.0) containing 10 mM NaCl for 20 min at 37 °C. The reaction was stopped by the addition of 500 µL 3,5-dinitrosalicylic acid (DNS) and heating the tubes in a boiling water bath for 5 min. The absorbance was determined at 540 nm by a spectrophotometer (BioMateTM-3, Thermo Scientific, Alpha Numerix, Webster, NY, USA). One amylase unit was defined as the amount of enzyme required to release 1 mM maltose/min at 37 °C under the given assay conditions. The results were carried out as a mean of three readings with an estimated margin of error of ±10% (mean ± standard error). 

### 3.7. Protein Concentration Determination

The total protein concentration of the samples was measured by the bicinchoninic acid assay [25] which was carried out in a microtiter plate by adding 0.05 mL of sample to 0.02 mL of bicinchoninic acid as working reagent. Consequently, the mixture was incubated at 37 °C for 30 min. A set of blank solutions was prepared containing same amount of particular diluted phase solutions without any enzyme sample. A set of blank solutions was prepared due to minimize any interference of the polymer and salt which containing the same amount of particular diluted phase solutions without the enzyme samples as a comparison to the enzyme samples for each system and absorbance of each of them were measured at 562 nm.

### 3.8. Acetone Precipitation

Acetone precipitation was employed before BCA assays to decrease the interference of thermo-separating polymer on the samples. A 1 mL of sample was added to 4 mL of acetone in a micro-centrifuge tube (5 mL). The mixture was vortexed to obtain a homogeneous solution, then the sample was transferred to a freezer and kept at −30 °C for 1 h. After incubation, the sample was centrifuged at 12,000 rpm for 10 min at 4 °C. The supernatant was removed and 1 mL of deionised water was added to the precipitated protein pellet to redissolve the protein pellet and achieve a protein solution. The BCA assay was subsequently performed on the protein solution [26]. 

### 3.9. Calculation of Partition Parameters

Presented below Equation (2) is the partition coefficient (K) of the amylase which was determined as the ratio of the amylase activity in the two phases [Equation (2)]:

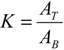
(2)
where A_T_ and A_B_ are the amylase activities in u/mL in the top and bottom phases, respectively. As presented [Equation (3)], Selectivity (S) represents the ratio of the amylase enzyme partition coefficient (*Ke*) to the protein partition coefficient (*Kp*):

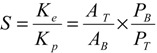
(3)
where K_e _and K_p _are the ratios of the amylase enzyme and protein concentrations found in each phase, respectively. A_T_ and A_B_ are the amylase activities (in units/mL) seen in the top phase and the bottom phases, respectively. P_T _and P_B_ are the total protein concentration (in mg/mL) observed in the top and bottom phases, respectively.

The top phase purification-fold (P_FT_) was obtained as the ratio of the specific activity of the amylase observed in the top phase to the initial amylase -specific activity found in the crude extract [Equation (4)]:

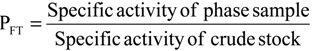
(4)
where specific activity is the ratio of the amylase activity to the total protein concentration of the sample. Also, the yield of the amylase obtained in the top phase was calculated using Equation (5):

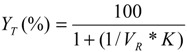
(5)
where V_R_ is the volume of top phase to bottom phase and obtained with graduated centrifuge tubes [Equation (6)]. Volume ratio is used to determine the concentration of proteins and yield or recovery of the enzyme in each phase [27]:

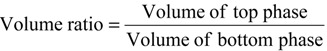
(6)


### 3.10. Determination of Purity of Amylase Using (SDS-PAGE)

SDS-PAGE was performed to analyze the purity of purified amylase utilising an electrophoresis unit (Bio-Rad, Foster, CA, USA). The electrophoresis process was run at 110 V and 36 mA for 75 min and on completion of the run, a solution of 0.05% (*v/v*) Coomassie^®^ Brilliant Blue G-250, 30% (*v/v*) methanol and 10% (*v/v*) acetic acid, was used for staining the gel [28]. Protein bands were observed after destaining by employing the same buffer without Coomassie^®^ Brilliant Blues.

### 3.11. Statistical Design and Analysis

All the experiments were organized using a completely randomized design (CRD). All assays were carried out in triplicate in the study. The analysis of the experimental data with two-way analysis of variance (ANOVA) was done, followed by fisher multiple comparison test at *p* < 0.05. Least significant difference test (LSD) was used to determine differences that were significant among the samples and all the data were expressed as mean ± standard deviation (SD) or standard error at a 90% confidence level (10% error) of independent trials. The Minitab software 14 (Minitab Inc., State college, PA, USA) was used to perform the data analysis. 

## 4. Conclusions

In this study, the effect of molecular weight of EOPO thermo-seperating polymers, volume ratio, crude load feedstock, and pH was evaluated due to optimization of ATPS employed for future recycling experiments. The optimum conditions were obtained using EOPO 2500 and 2-propanol with 25% crude load, and volume ratio 1.94 at pH 5.0. Under the optimized conditions, a high yield of 96.6% and a 14.3-fold purification of purified amylase were obtained. More than 90% of copolymer (EOPO 2500) was recycled from the ATPS based on thermo-separating polymer. The direct recovery of amylase from pitaya waste employing the ATPS based on thermo-separating polymers was shown to be a successful method for purification of the enzyme from fruit sources. This study also indicated that the phase components can be recycled and reused, resulting in this procedure being more efficient, economical and providing a mild environment, especially for the large scale production of the enzyme from fruit waste. 

## Abbreviations

ATPS: Aqueous Two Phase System
PEG: Polyethylene Glycol
EOPO: Ethylene Oxide-Propylene Oxide
BSA: Bovine Serum Albumin
DNS: 3,5-dinitrosalicylic acid
SDS-PAGE: Sodium Dodecyl Sulfate Gel Electrophoresis
CRD: Completely Randomized Design
ANOVA: Analysis of Variance
LSD: Least Significant Difference
SD: Standard Deviation
